# Deriving A Drinking Water Guideline for A Non-Carcinogenic Contaminant: The Case of Manganese

**DOI:** 10.3390/ijerph15061293

**Published:** 2018-06-20

**Authors:** Mathieu Valcke, Marie-Hélène Bourgault, Sami Haddad, Michèle Bouchard, Denis Gauvin, Patrick Levallois

**Affiliations:** 1 Direction de la Santé Environnementale et de la Toxicologie, Institut National de Santé Publique du Québec, 945 Avenue Wolfe, Québec, QC G1V 5B3, Canada; mathieu.valcke@inspq.qc.ca (M.V.); marie-helene.bourgault@inspq.qc.ca (M.-H.B.); denis.gauvin@inspq.qc.ca (D.G.); 2 Department of Environmental and Occupational health, École de Santé Publique, C.P. 6128, Succursale Centre-Ville, Montréal, QC H3C 3J7, Canada; sami.haddad@umontreal.ca (S.H.); michele.bouchard@umontreal.ca (M.B.); 3 Department of Social and Preventive Medicine, Faculté de Médecine, Pavillon Ferdinand-Vandry, 1050 Avenue de la Médecine Local 00241, Université Laval, Québec, QC G1V 0A6, Canada

**Keywords:** drinking water, inorganic manganese, health-based guideline, infants

## Abstract

Manganese is a natural contaminant of water sources. It is an essential oligo-element, which may exert toxicity at high doses, particularly via inhalation. Its toxicity by the oral route is less known, but epidemiological and experimental studies tend to support its neurodevelopmental toxicity in infants and children. This paper describes the method used by a middle-size public health institution to derive a Drinking Water Guideline (DWG) for manganese. After reviewing the work done by major public health institutions, authors confirmed the use of experimental data to derive a point-of-departure (POD) of 25 mg of manganese/kg/day, based on neurodevelopmental effects on pup rats. Then, a total uncertainty factor of 450 was applied to calculate a Toxicological Reference Value (TRV) of 55 µg/kg/day. The final DWG proposed for manganese is 60 µg/L and is based on a relative source contribution (RSC) of water of 20% and an infant drinking scenario of 182 mL/kg of body weight (BW) of water (95th percentile of the ingestion rate distribution for 0–6 months). Despite its limitations, e.g., starting with the work done by other agencies, such an approach demonstrates in a transparent way the rationale and challenging choices made by regulators when deriving a DWG.

## 1. Introduction

Manganese is a metal mostly occurring naturally in the environment. Food is the main source of exposure for humans, but water may sometimes become a significant source. In particular, high levels of manganese may be found in surface or groundwater, occurring in aerobic or low oxidation conditions [1]. Inorganic manganese is considered an essential element for human beings, despite few reported cases of clinical deficiency [2]. It has a well-known toxicity in workers exposed by inhalation, leading to neurotoxic effects and Parkinson-like symptoms, sometimes called manganism [3]. However, its toxicity by the oral route has long been considered low, particularly due to homeostasis control of its excretion.

Very few epidemiological studies were conducted on manganese exposure from drinking water in adults. They had limited designs and gave mixed results [4,5]. However, several studies with better designs have since then reported possible negative impact on the neurodevelopment of infants and young children [6,7,8,9]. Infants might be particularly vulnerable to overexposure due to their greater gastrointestinal absorption and immaturity of their homeostatic control of bile excretion [2,10]. Therefore, growing concerns with regard to manganese neurotoxicity are being expressed [10,11].

Until now, few jurisdictions have proposed a drinking water quality criteria for manganese based on health effects. The last version of the WHO guidelines did not report any formal guideline for manganese, due to the fact aesthetic acceptability problems usually occur before health concerns [1]. The USEPA has proposed a lifetime health advisory of 300 µg/L based on a dietary daily intake of 10 mg for adults and 20% contribution for drinking water [12]. The same value was also recommended for infants younger than 6 months for an acute exposure of 10 days, contrary to 1 mg/L for older children and adults, because of concerns of possible overexposure of infants [12]. More recently, the Minnesota Department of Health has proposed a short term Health-Based Value of 100 µg/L [13]. In doing so, the possible higher risk for the neurodevelopment of infants bottle fed with plain tap water or given reconstituted formula is specifically targeted. Indeed, this guideline is based on cognitive and behavioral effects found in newborn rats exposed to manganese in water [14] and accounts for infant-specific body weight-adjusted drinking water ingestion rate.

Such a guideline brings out the growing concerns of public health institutions with regard to the particular susceptibility of young infants to manganese, not only from the perspective of the health effect but also of the increase exposure, also highlighted by Goeden [15] (in this *IJERPH* special issue). Given the presence of manganese in several groundwater sources in Quebec [16,17] and the absence of any official health based standard for this parameter in Quebec and Canada, the Institut de santé publique du Québec (INSPQ, Québec, QC, Canada) was asked by the Quebec Ministry of Healh and Social Services to propose a health-based guideline value for manganese in drinking water. The objective of this paper is to describe a systematic method applied by the INSPQ to derive a Drinking Water Guideline (DWG) for the non-carcinogenic contaminant that is manganese.

## 2. Methodology

We present here the method used by the INSPQ to derive its health-based drinking water guideline for manganese in Quebec [18]. It relies mainly on the critical use of assessments already made available by public health institutions around the world for manganese. The approach includes four mains steps, namely: (1) identification of the existing relevant toxicological reference values (TRV) derived by these institutions in order to select the most appropriate point of departure (POD); (2) determination of the required uncertainty factors (UF) based on available data rather than default values if possible; (3) application of the required UF to divide the POD determined in step 1 and (4) consideration of the relative source contribution and drinking water ingestion rate in order to compute the final numerical value of the DWG.

### 2.1. Inventory of Available TRVs for Selection of the Most Appropriate POD

TRVs for manganese were searched among the following sources: Health Canada’s Drinking Water Documents [19], US EPA’s IRIS and Drinking Water Health advisoires [12,20], CalEPA’s Public Health Goals [21], the Human Health-Based Water Guidances from the Minnesota Department of Health (MDH, St. Paul, MN, USA) [22], WHO’s Drinking Water Guidelines [1], ATSDR’s Minimum Risk Levels (MRL)s [23], and the Advices for Drinking Water Quality from the French Agency for Food, Environment and Occupational Health & Safety (ANSES, Maisons-Alfort, France) [24]. These represent the most commonly cited public health institutions with regard to the determination of populationnal TRVs for environmental contaminants. Not only official positions are retained at this step, but also provisional guidelines and draft documents when they are publicly available.

The POD that could be identified for manganese from these institution reviews were critically analyzed based on the following criteria: (1) the relevance of the exposure route to the targeted guideline; (2) the robustness of the original dose-response curve that have lead to the determination of the POD; (3) the correspondence with the most sensitive adverse health effect.

Precisely, given that the DWG purpose is to limit exposure resulting from the ingestion of drinking water, TRV for the oral route are to be focused on. In the case of animal studies, administration via drinking water rather than food or gavage is to be privileged, but epidemiological data are prioritized over animal data when both types are considered of equivalent quality and robustness. With regard specifically to the POD, benchmark doses are to be prioritized over NOAELs, as they include all the available information provided by the dose-response curve, contrary to a single experimental point, such as the NOAEL [25]. If none of these critical doses are available, LOAELs can be used. Finally, should several PODs appear relevant based on the preceding criteria, the one corresponding to the most sensitive effect, that is the lowest, is chosen.

### 2.2. Review and Application of The Required Uncertainty Factors

The application of uncertainty factors (UF) follows the general principles edicted by the US EPA [26]. Such factors are applied to compensate for the uncertainty resulting from the use of a POD that has been determined in conditions that differ from the conditions to which the targeted TRV applies [27,28,29]. Thus, UF for animal-to-human extrapolation (UF_A_), interhuman variability (UF_H_), subchronic-to-chronic extrapolation (UF_S_), LOAEL-to-NOAEL extrapolation (UF_L_) are considered. All these UFs are usually attributed a 10 default value. Additionally, the available studies, among which the one used for the determination of the POD is included, must comprise at least two chronic toxicity studies and two developmental toxicity studies by the relevant exposure route, in a rodent and non-rodent mammal species, as well as a multigenerational reproduction study. Otherwise, an additional factor is applied to account for the deficiency in the database (UF_D_) [27,28,29]. The approach considered here implies that the value attributed to UF_D_ is either 3, if one or two of the aforementioned studies is missing, or 10 if more than two these studies are missing, or if the pattern of the dose-response curve is unclear, if suspected evidences of epigenetic carcinogenicity are present or any other justification that cannot be covered by the above-mentioned UFs. Once all the required UFs are identified, a so-called “customized” TRV can be calculated, as the POD divided by the product of each selected UF.

In accordance with the WHO/IPCS and US EPA guidances on the application of chemical-specific or data-derived extrapolation adjustment factors [30,31,32], it is recommended to consider the possible replacement of the 10-fold default value generally applied UF_A_ and UF_H_ by numerical values that are computed from data relevant to the chemical of interest. This is proposed under the premise that the default 10-fold value in fact corresponds to two components, one for toxicodynamic (TD) and one for toxicokinetic (TK) variability.

In the case of manganese, available data only allow this replacement for UF_H_, for which the default TK and TD component values are being attributed the value of square root of 10, that is 3.16 [30,31,32]. First, the comparison between average and sensitive individuals in both species was based on available experimental data in rats and population biomonitoring data in humans [33,34,35,36]. This allowed to evaluate whether or not the interindividual TK variability is greater in humans than in rats. Such evaluation was necessary given that the POD retained results from a study conducted in neonate rats (see Results), thus suggesting that the presumed increased sensitivity of newborn individuals, as compared to adults, is intrinsically accounted for by this POD selection. The magnitude of the interindividual TK variability that is possibly not accounted for by this POD selection was then evaluated. Indeed, it cannot be excluded that some TK variability remains within the sensitive human subpopulation itself, while it is apparently virtually limited in rats [33]. Thus, total blood Mn concentrations measured in several human subpopulations presumed more sensitive to the effect of Mn were taken from the literature, and the magnitude of the TK variability computed as prescribed by the WHO/IPCS [30] and US EPA [31], and total UF_H_ was calculated by multiplying resulting TK and TD component accordingly.

### 2.3. Caclulation of the Drinking Water Health-Based Guideline

The drinking water health-based guideline for manganese is calculated based on the oral TRV determined above following the state-of-the-art approach for non-carcinogenic effects [1,37,38,39], given that the critical effect of manganese is of neurotoxic nature [3,11,40]. Precisely, equation 1 show that the relative source contribution (RSC), the body weight (BW) and the drinking water ingestion rate (IR) are factored in for the guideline calculation:

DWG = (TRV × RSC × BW)/IR(1)

where, DWG is in mg/L, TRV is in mg/kg/day, RSC is unitless, BW is in kg, and IR is in L/day.

The relative source contribution (RSC) of drinking water corresponds to a fraction of the TRV to which the exposure resulting from the use of drinking water can contribute. Indeed, it is necessary from a public health perspective to ensure that this contribution does not result into exceeding the TRV if summed to the exposure resulting from the non-drinking water potential sources [1,41,42,43,44]. Given the default assumption that total exposure to an environmental contaminant may come from 5 medias (drinking water, air, soil, food and household products; [1,41]), it is considered that up to one fifth of this total exposure can be attributed to drinking water, thus triggering a default 20% RSC value for all age groups. In certain circumstances, for which further details are provided in Supplemental materials, an RSC value that differ from this 20% default can be chosen. That is, a RSC greater than 20% is justifiable when drinking water is believed to be the main or the only source of exposure, while an RSC lower than 20% can be attributed when it is estimated that the other exposure sources than drinking water, notably food, may contribute in total, to more than 80% of the TRV [1,41,42,43,44]. Manganese being an essential element and given that its main exposure source is in fact food, its RSC was herein validated based on data of its concentrations found in cow or soya milk formula, which constitutes the main manganese dietary exposure source for non-breast-fed neonates.

The choice of the values that are used in Equation (1) for BW and IR is driven by three considerations, namely (1) the period of life during which the exposure that has allowed the determination of the POD has occurred; (2) the concern for protecting the most sensitive individuals; and (3) making sure that the DWG is adequately protective for the high-end consumers of drinking water. The specific values chosen in the case of manganese are detailed and justified in Results, hereafter.

## 3. Results

### 3.1. Available Oral TRV for Manganese

The oral TRVs adopted for manganese by the various institutions investigated are detailed in Table 1. They originate either from human or animal data. With the exception of MDH, every reviewed institution have determined an oral TRV that applies to chronic exposure of the general population. In the case of MDH, two TRV were determined, namely one for the chronic exposure of the general population, which in fact is the same as the US EPA’s, and another one for exposure occuring in the first year of life (short-term TRV). Oral TRV chosen by the US EPA, WHO and ATSDR are based on human data, whereas the short-term TRV derived by MDH is based on animal data.

In addition to the institutions indicated in Table 1, Health Canada has also recently proposed, as part of a public consultation, an oral TRV of 25 µg/kg BW/day based on a LOAEL of 25 mg/kg/day divided by a composite UF of 1000 [19]. This value has not been officialy adopted to date, but is still considered for the purpose of the present work, as it counts among the most recent documents published on manganese by a recognized public health organization.

### 3.2. Determination of the Most Relevant POD

The TRV derived by the US EPA, WHO and ATSDR are all based on the upper bound estimated daily dietary intake of manganese in the average vegetarian adult, determined as safe by various authors after further analyses [2,46,47,48,49,50]. However, this TRV is not determined based on a toxicological evaluation [10]. Moreover, either no data related to dietary manganese exposure are presented in the cited sources, either these sources refer to other studies with limited data and for which there was no focus on health outcome. Also noteworthy, these studies have been published back to at least the 80’s, and as early as the 60’s. For these reasons, the human data-derived POD from the US EPA, WHO and ATSDR is not retained here for the DWG derivation for manganese.

Conversely, considering the increasing weight-of-evidence towards infant neurotoxicity of manganese in drinking water as the critical effect of interest, MDH and Health Canada have evaluated the possibility to determine such guideline based on corresponding epidemiological data [13,19]. However, due to important limitations of available studies in this regard, both organizations have considered that animal data are to be privileged. The INSPQ agrees with this evaluation. Thus, a POD corresponding to a LOAEL of 25 mg/kg/day for neurobehavioral, motor and cognitive effects obtained in juvenile rats exposed on post-natal days 1–21 (*per os*), or longer (via drinking water) to doses of 0, 25 or 50 mg/kg/day of manganese under the form of manganese chloride (MnCl_2_·4H_2_O), was retained by both institutions [13,19]. This POD results from a series of three studies conducted by the same research team [14,51,52]. In each one of these studies, at least one adverse effect occurred at exposure doses of 25 mg/kg/day on post-natal days 1–21 [14,51] or during the entire 54 weeks following birth [52], thus corresponding to a LOAEL (Table 2).

Given the good quality of all three studies listed in Table 2 (precise experimental protocols, control of confounding’s, justification of the chosen exposure doses), this neurodevelopmental LOAEL of 25 mg of manganese/kg/day was chosen as a POD for the current DWG derivation. Although other LOAEL lower than those POD have been proposed in some studies (reviewed by Health Canada [19], see Supplemental materials), important limitations preclude their use in the present context. In particular, these studies often included a single experimental dose or involved controls that were not readily comparable to the exposed group, which equals to testing a single experimental dose.

### 3.3. Application of Uncertainty Factors

#### 3.3.1. Default 10-Fold Value or not Applied (UF = 1) Uncertainty Factors

Given that no available data suggest a value that differs from the default 10-fold uncertainty factor for animal-to-human extrapolation (UF_A_), it was retained for the current assessment.

The POD retained reflects an exposure that has occurred over a short period of time during the first three weeks of life of the experimental pup rats, and to which are associated neurodevelopmental effects occurring up to three months later. But prolonged exposure throughout lifetime did not result in a different LOAEL [52]. Likely, this brings out the evidence of a mechanism of toxicity that involves a window of susceptibility, and that the adverse effect that may occur following exposure during this period is in fact the most sensitive one. Therefore, even though exposure may persist for longer duration, it will likely not trigger another adverse effect than the one associated to that specific window of susceptibility. This is further evidenced by the fact that LOAELs observed in chronic animal studies for other adverse effect than the one considered here almost always exceeded 25 mg/kg/day [3]. Two exceptions are the studies of Gupta et al. [53] and Ishizuka et al. [54] which however the number of individuals exposed was very low (4 rhesus monkeys and 2 mice, respectively), providing evidences of very limited robustness. Thus, no subchronic-to-chronic uncertainty (UF_S_) factor was applied here.

#### 3.3.2. Uncertainty Factors Different from the Default 10-Fold Value

Uncertainty factors that were applied and exhibit a magnitude that differ from the default 10-fold value include those for human variability (UF_H_), for LOAEL-to-NOAEL extrapolation (UF_L_) and for the incomplete database (UF_D_).

##### Human Variability

Experimental kinetic data on manganese in rats suggest that more than 80% of an administered dose in neonate rats is retained as body burden [33] whereas this fraction is only about 5% in pup rats and stays around this level until adulthood [34], suggesting a 16-fold variations between identified sensitive and average rat. This is attributable to a reduce homeostatic capacity in the first few weeks of life [33,34]. In humans, Japanese data were available with regard to measures specifically conducted in newborns. Indeed, Mizogushi et al. [35] reported an arithmetic mean (± standard deviation (SD)) total blood concentration of manganese of 56.4 ± 16.4 µg/L in this subpopulation (*n* = 14), based on which a 95th percentile value of 83.4 µg/L can be computed as [mean value + (1.64 × SD)], assuming lognormal distribution. In comparison, the mean concentration in subjects aged 1–11 years (*n* = 36) was 14.8 µg/L. Considering the expected difference between an arithmetic mean and a geometric mean in a population distribution, this latter value compares rather well with the geometric mean concentration observed in children aged 6–11 from the Canadian Health Measure Survey (CHMS), that is 11 µg/L [36]. Besides, mean total blood manganese levels barely vary through lifetime according to CHMS, as values between 9.1 and 10 µg/L were obtained in the participants aged from 12 to 79 years old. From these elements, it can be suggested that the magnitude of the variability of manganese levels is comparable between Japanese and Canadian populations. Therefore, using the Canadian mean adult value of 9.8 µg/L for 6 to 79 years old [36], and under the WHO/IPCS approach for the calculation of chemical-specific adjustment factor [30], the magnitude of TK variability for manganese in humans can be approximated as the ratio between the 95th percentile in sensitive individuals, here newborn japanese over the median in adults, that is the Canadian value, yielding 83.4/9.8 ≈ 9. This ratio appears rather conservative, as the corresponding ratio between newborn and older Japanese children would have yield a value of ≈ 5.6 (84.5/14.8). Ratio values of 3 or less were observed by Park et al. [55] as well as Alarcon et al. [56] in respectively Korean and Venezuelan infants. Even when considering reported manganese levels in pregnant women, umbilical cord and neonates, including in 12 comparison studies detailed by Huang et al. [57], such ratio remains below 10 [57,58].

All in all, from the data mentioned in the preceding paragraph suggest that the magnitude of the interindividual TK variability in rats is comparable, or even slightly greater, than that calculated from human data. Although this assertion is made based on a quite limited number of measures (11–24 per dose in rats depending on the study and 14 in humans), which limits their generalizability to entire animal or human populations, it appears reasonable to propose that a major part of interindividual TK variability is presumably accounted for by the use of a POD determined in neonate rats, and the TK component of UF_H_ can be diminished below its default 3.16 value. Also, literature data on manganese TK variability within several presumably sensitive human subpopulations are presented in Table S1. It can be seen that following the WHO/IPCS approach, [30], the computed interindividual variability in toxicokinetics corresponds to a 1.7-fold magnitude in average, within the most sensitive human individuals. This thus replaces the 3.16 default value for interindividual TK variability factor and multiplies the other 3.16 default value for interindividual TD, yielding a resulting rounded UF_H_ value of 5.

##### LOAEL-to-NOAEL Extrapolation

Based on ATSDR’s compilation, both LOAEL and NOAEL values can be identified among several of the reviewed experimental studies in neonate rodents, which covered a wide array of such effects [14,51,52,59,60,61,62]. In each one of these studies, the NOAEL is at the most 3 times lower than the LOAEL; therefore, a 3-fold LOAEL-to-NOAEL UF_L_ is chosen here.

##### Database Deficiencies

The available relevant studies on manganese detailed by Health Canada [19] include all of the required studies as per the state-of-the art determination of TRV (see Methods) [26,27,28,29]. Therefore, no UF_D_ would be needed under this aspect. However, some of the studies that were not retained here due to the weaknesses mentioned in 3.2, still suggest a neurodevelopmental LOAEL that could be lower than the 25 mg/kg/day value used herein (See Supplemental Materials). In particular, the studies from Dorman et al. [61] and Brennemann et al. [60] suggest LOAEL values of 11 and 22 mg/kg/day, but the toxicological significance of the adverse effects considered in these studies (increased reaction to an acoustic stimulus, and increased motor skills, respectively) is unclear. Still, a 3-fold UF_D_ is added in the current analysis at this step to compensate for the uncertainty added by the existence of lower LOAEL values that were not retained for the purpose of the current assessment.

### 3.4. Determination of a Customized TRV

Based on the POD retained in 3.2 (LOAEL of 25 mg/kg/day) and the product of every uncertainty factors applied as justified in Section 3.3 (UF_H_ × UF_A_ × UF_SC_ × UF_L_ × UF_DB_ = 5 × 10 × 1 × 3 × 3 = 450), the TRV calculated in this assessment is 25/450 = 55 µg/kg/day.

### 3.5. Consideration of the Relative Source Contribution and Drinking Water Ingestion Rate

#### 3.5.1. Relative Source Contribution

Elevated manganese concentrations, up to the range of 300 à 400 µg/L, had been found in milk formula, in particular based on soya milk [10,63]. A Canadian study has examined manganese concentrations in various drinks destinated to neonates and infants [64]. Despite limited sample size, a mean manganese concentration of 100 ± 50 µg/L was measured in a cow milk-based formula. Corresponding value for soya milk-based formula was 340 ± 110 µg/L.

Although present at roughly 3.4 times greater concentrations, manganese in soya milk-based formula is up to 8 times less bioavailable than in cow milk-based formula; this is attributed in part to the presence in soya milk of other chemicals (e.g., phitic acid) that compete with manganese for GI absorption [65]. Overall, it appears reasonable to state that cow milk-based formula represents a potential for greater internal manganese dose. Therefore, based on a 95th percentile manganese concentration of 182 µg/L in cow milk-based formula computed under the same default statistical considerations as those described in 3.3.2., an exposure dose of 33 µg/kg/day can be calculated. This figure corresponds to 60% of the TRV determined above (55 µg/kg/day) and leaves a margin of 40% of the TRV to every non-dietary sources of manganese exposure, drinking water being only one of them. Else, the available data do not suggest that dietary and other non-drinking water sources contribute to more than 80% of the total exposure. Overall then, under the premise of parting equally the above-mentioned 40% contribution between drinking water on the one hand, and every other non-dietary sources on the other hand, the default value of 20% can be retained here for DWG derivation.

#### 3.5.2. Body Weight (BW) and Drinking Water Ingestion Rate (ING)

Given that the TRV determined above for manganese is based on a POD determined under the consideration of a specific period of susceptibility, the values considered to the parameters related to the drinking water contact rate are deemed to reflect this specific time period. Therefore, the mean BW and 95th percentile value of ING for the infants aged ≤ 6 months for the Quebec population, described elsewhere [66], are being used for the DWG calculation. This corresponds to 6.7 kg and 1.22 L/day respectively, for a BW-adjusted equivalent ingestion rate of 0.182 L/kg/day. Other elements considered by the present authors with regard to the choice of the appropriate BW-adjusted DW ingestion rate are presented in Supplemental materials.

### 3.6. Calculation of the Drinking Water Guideline

Applying Equation (1) above to the TRV calculated in 3.4, the DWG for manganese is 61 µg/L, rounded to 60 µg/L and based on a 20% RSC, a BW of 6.7 kg and a drinking water ingestion rate of 1.22 L/day. This guideline is more stringent than similar guidelines proposed by other public health institutions around the world, as shown by Table 3.

## 4. Discussion

The objective of this work was to derive a health-base DWG for manganese and provide a demonstration of the procedure used by a public health agency of a midsize jurisdiction such as the province of Quebec, that is INSPQ, for such activity. The work is mainly based on a systematic analysis of already existing guidelines determined by other public health institutions, with a focus on the rationale and critical points determined by these institutions to justify their methodological choices. Such an approach presents the advantage in that by building on existing work, it allows efficient process of guideline derivation while optimizing often limited time and human resources in middle size public health institutions. The DWG obtained here for manganese is 61 µg/L, rounded to 60 µg/L, and was derived by focusing specifically on the concern of protecting the most vulnerable population, that is children aged < 1 year old, not only by selecting the lowest relevant POD available and applying the required UF, but also by considering a BW and drinking water ingestion rate that corresponds specifically to this age group. Thus, the resulting guideline accounts for the greater drinking water ingestion rate in neonates as compared to adults, on a BW basis. It therefore addresses the concerns raised by Goeden [15].

Despite the fact that there are lower guidelines for manganese in drinking water based on esthetical considerations, that is 50 µg/L and currently projected to be lowered to 20 µg/L [19,68,69], the present health-based guideline is more stringent than corresponding health guidelines around the world, as the next most severe guideline is MDH’s Risk Assessment Advice of 100 µg/L. Reasons for such discrepancy can be identified from the values taken by the different variables entering in the guideline calculation, which are detailed in Table 3.

The main source of greater conservatism in the current assessment as compared to the US EPA and WHO mainly stems from the choice of the POD, an animal one that triggers the application of several UFs, rather than the epidemiological POD with almost no UF applied. Indeed, the former appears more justifiable given that in the latter case, the human data that were not evaluated from the toxicological point of view, but rather the nutritional one [10].

The choice made here of the neurodevelopmental animal LOAEL of 25 mg/kg/day is also justified based on the following four elements:

(1) Neurotoxic effects of ingested manganese are the most sensitive ones based on the array of the toxicological tests listed in Table 2. Besides, this effect was observed in pup rodents at BW-adjusted exposure doses that are lower than those observed in other rodent groups (post weaning or adult animals) exposed chronically or subchronically to manganese [3]. Such sensitivity in rodent pups appears related to the immaturity of their homeostatic regulation system, with the consequent maximal absorption and minimal, if not absent, excretion during the first 15 to 18 postnatal days [33,70,71,72].
(2) Because of the uncertainties associated with the characterization of exposure and the role of confounders in epidemiological studies, corresponding data with regard to cognitive, motor and behavioral effects [19,73] are difficult to use for quantitative risk assessment such as the derivation of a health-based drinking water guideline.
(3) Although non-human primates would constitute the most appropriate toxicological model in order to characterize the toxicity of ingested manganese in humans [74,75], the available data do not appear sufficiently robust to do so. Indeed, such data result from experimental designs that either involve a single tested dose or a manganese exposure occurring via the intravenous route, or lacks comparable control experimental groups [53,75,76,77,78,79,80,81,82].
(4) The mechanism of action of manganese neurotoxicity through the oral exposure route is not fully elucidated. It is likely multi-process, including perturbation of dopamine transmission [40]. Given that such perturbation has been observed in both rodents and primates exposed to manganese [74], using a rat-based LOAEL to determine a DWG in humans appears relevant.

Once the choice of relying on animal data rather than human data has been made, differences in the application of UF contribute to derive a different guideline than MDH and Health Canada. However, at the end and as shown in Table 3, the composite UF of 450 positions itself in between Health Canada composite UF (1000) and MDH one (300). As a result, the TRV obtained (55 µg/kg/day) also lies midway between these two institution values (25 and 83 µg/kg/day). These numbers can be analyzed in light of the essential nature of manganese by comparison with the minimum recommended intakes of the Institute of Medicine (IOM). The TRV obtained here is significantly greater than the minimum intake recommended for neonates aged ≤ 6 months (≈0.5 µg/kg/day) but may be lower than, or equivalent to, this recommendation for other age groups, in particular children aged 1–3 years (80 µg/kg/day) and 4–8 years (55 µg/kg/day) [2]. However, IOM recommended intakes are based on real exposure data that are deemed corresponding to the minimum intakes required. Possibly, the true required intake is much lower.

A source of greater conservatism herein is the application of 3-fold UF_DB_ to account for the fact that some lower LOAEL values—although apparently less robust than the POD selected here—could have been used (See Supplemental Materials). This factor is not applied by any other public health institution. Conversely, in some instances, less severe UF values were used. This is the case for UF_H_, as a 5-fold factor was applied here contrary to the default 10-fold value applied by both MDH and Health Canada. As clearly demonstrated in Section 3.3.2, available data support the application of a less-than-ten-fold factor for UF_H_. In fact, MDH and Health Canada both argue that a significant variability is observed in manganese metabolism in rats and thus such variability need to be accounted for when extrapolating to animals (personnal communication with Health Canada and MDH). However, it is the current authors position that this variability either leans towards greater variability in rats than humans, or that the toxicokinetically more sensitive individuals were accounted for by the experimental design leading to the selected animal POD. In such situation, it can be argued that only the toxicodynamic component of the UF_H_, that is 3.16, need to be accounted for. Since some residual uncertainty still resides in assessing human variability from animal variability data, it appears reasonable that such situation triggers an UF_H_ no lower than 4, and the data-derived approach used here is coherent with this argument.

In support of the current assessment, both MDH and Health Canada have applied the default 10-fold animal-to-human extrapolation factor while neither institution applied any UF for the use of a less-than-chronic POD in guideline derivation. While MDH guideline specifically targets short-term exposure [13], this position appears well justified. Health Canada, for its part, argues that the consideration of a POD triggered by an exposure occurring during a restrained window of susceptibility, as can be concluded from the experimental design detailed in Table 2, suggests that the measured effect is likely more sensitive than any other (chronic) effect that may result from long-term exposure to manganese in drinking water and that as a result, no UF for subchronic to chronic extrapolation is required. Other coherent UF values include the 3-fold UF_L_ applied here like MDH and differently than the 10-fold default applied by Health Canada. This 3-fold value was also based on the review of the magnitude of the LOAEL/NOAEL ratios for a same given adverse effect, detailed by ATSDR (see also Supplemental Material). However, MDH argument differs in that it qualifies as subtle the neurological adverse effect and thus does not require a 10-fold factor.

The attribution of a RSC of 20% rather than 50% contributes significantly to the derivation of a lower guideline than MDH and Health Canada. Both institutions have justified this 50% value by the fact that neonates are mainly exposed to manganese from two sources, namely drinking water and food, each of which 50% of the overall contribution is attributed. Although this is based on some rationale, the careful analysis of the available quantitative data suggests otherwise with regard to the magnitude of this factor, as the 50% value retained by these institutions appear too high. Indeed, if 50% of the TRV of 55 µg/kg/day calculated in Section 3.4 was attributed to drinking water, the potential is real for some infants to be exposed to a total dose exceeding it. Given the 33 µg/kg figure calculated in Section 3.5.1, this corresponds to 60% of the TRV as resulting from ingestion of milk formula, leaving at the most a RSC for drinking water that should not be greater than 40%.

In this regard, it is noteworthy that Krishnan and Carrier [20] describe the US EPA methodology for deriving values that depart from default for this factor, based on available data on the presence of the contaminant of interest in the various environmental medias. Thus, an RSC lower than 20% can be required if it is estimated that the other exposure sources than drinking water may contribute, at the total, to more than 80% of the TRV [1,31], as allowing as much as 20% of RSC to drinking water in this case would undesirably translate into a total exposure that theoretically exceeds the threshold of potential effect. Conversely, an RSC greater than 20% can be envisaged when it is known that drinking water is likely the sole (e.g., up to 80%) or at least the main (e.g., at 50%) contributor to the total human exposure [1,26,28,30,31], for instance because a chemical is added for water treatment purposes (e.g., trihalomethanes, trihaloacetic acids). However, the data-derived approach implies that for a situation in which the drinking water is more contaminated than the other medium, its RSC can be greater than 20% and as a result, the guideline more permissible for more contaminated media than for less contaminated ones. Although this can be justifiable from a risk management perspective, it is more hardly so when reasoning strictly from the point of view of protecting human health. Besides, the IOM stated that, for infants, the sole source of manganese as essential element should come from dietary sources [2], therefore excluding any presence in drinking water. Obviously, this recommendation was made at a time where not many toxicological data on manganese were available. But the default 20% value was still retained here, and the data-derived approach proved to be useful in demonstrating that the 50% figure appears too permissive.

One uncertainty that is worth mentionning with regard to the DWG determined here relates to the fact that manganese speciation is sensitive to environmental conditions and drinking water sources may vary considerably with regard to their physicochemical environment. Thus it cannot be excluded that the population, for which the DWG aims at regulating its manganese exposure, in fact is exposed to chemical forms of manganese that are different than the one that was used in the study from which originates the POD used herein. Likewise, the dose-response relationship could be different. However, the manganese chloride that was used in the chosen POD study is very soluble and is ionized as Mn^2+^ in water, which happened also to be the vehicle of administration in this study. Besides, epidemiological studies that brought out concerns for the neurodevelopmental toxicity of manganese (see Introduction) have been conducted on population fed by groundwaters and it is therefore reasonable to assume that most manganese was also present as Mn^2+^. Overall then, the impact of the uncertainty associated with the possible variations of the dose-response relationship according to the chemical form of manganese appears low with regard to the adequacy of the DWG proposed herein.

A limitation of the method presented here that needs to be acknowledged is that a complete literature review of all toxicological and epidemiological data available on manganese toxicity was not performed, but rather a critical analysis of previous work done by other Agencies was done [12,13,19]. In particular, the POD for this risk assessment was based on a critical analysis of papers, which were already selected and reviewed by those agencies. This approach was chosen in response to limited resources but still appears appropriate in view of the credibility of these agencies and the fact that, at least for the MDH [13,45] and Health Canada [19], their reviews of the literature were quite recent. Should there have been no recent review available by credible agencies, a complete literature review would have been required, but only for the time period not covered by the previous reviews. Despite the fact that such an approach has some limitations (no replication of the original literature reviews), it allows middle-size agencies to do such work with limited resources and time constraints, which is not a negligible advantage from a public health perspective.

Finally, this DWG for manganese is based on a risk assessment conducted on the most vulnerable population to this contaminant: infants fed with formula reconstituted with drinking water. Normally, for convenience and simplicity of the communication message, it might be justified to propose such a guideline for the entire population exposed to excessive concentration of manganese in drinking water. However, because the homeostatis control for oral exposure is more developed in older children and adults, and cost of measures to be applied to reduce exposure (water treatment, changing the source of water., etc.), some juridictions will prefer to use a different DWG for older populations. This is the case of MDH who has recommended to use the USEPA Health advisory of 300 μg/L for older population (>1 year old) [13]. Water treatment technologies, both at the community and individual levels are available, but their implementation may represent additional financial costs and significant challenges for drinking water system managers. However, given the organoleptic inconveniences caused by the presence of manganese for exposed population, in particular staining of laundry and plumbing fixtures, and eventually taste problems, it might be justified to implement measures to reduce the presence of manganese in drinking water for the entire population. Thus, the organoleptic recommendation of 50 μg/L, as currently proposed by US-EPA [38] and WHO [68], or a lower one as the one proposed recently by Health Canada [19], might therefore be considered as a target to be achieved for the entire population. Our proposed DWG is not very different from these esthetical guidelines.

## 5. Conclusions

Deriving DWG is an important activity to protect public health [1]. However, the procedures used to derive a guideline are sometimes very technical and not available in current literature. A simple method used by a middle size institution (INSPQ, Québec, QC, Canada) using the critical review of work done by other institutions as its first input was thus presented here, following a step by step method to derive a guideline for non carcinogenic contaminants.

The proposed health based guideline proposed for manganese is based on the same animal POD as the one chosen by other institutions [13,19] but its numerical value differs by using different uncertainty factors, RSC of water to the TRV and water consumption. Globally, the proposed guidelines of 60 µg/L is still higher than the usual esthetical guidelines values for manganese (50 µg/L, projected to be lowered at 20 µg/L, [1,19]) but lower that other health based values [12,13,19]. The rationale applied to the choices made herein were exposed and compared to the choices of other institutions. This might help to open a transparent debate on the issues relative to deriving health based guidelines for drinking water.

## Figures and Tables

**Table 1:** Oral TRV recommended by various public health institutions.

Institution	POD (mg/kg/day)	Type of POD	Composite UF	Oral TRV (µg/kg/day)
TRV based on human data				
US EPA and MDH [12,20,45]	0.140 ^1^	NOAEL	1 or 3 ^2^	140 or 47 ^2^
WHO [1]	0.183 ^3^	NOAEL	3	61
ATSDR [3]	0.160	NOAEL	1	160 ^4^
TRV based on animal data				
MDH [13]	25	LOAEL	300	83 ^5^

^1^ Rounded value, based on a NOAEL of 10 mg/day for a 70 kg adult; ^2^ Uncertainty factor that applies only to non-dietary manganese exposure, including drinking water, for individuals older than 1 year-old. ^3^ WHO has selected a NOAEL of 11 mg/day for a 60 kg adult. ^4^ Interim guideline. ^5^ This TRV only applies to infants younger than 1 year old.

**Table 2:** Summary of the neurological effects observed in rats exposed orally to Mn following birth, at doses of 0 (control group), 25 and 50 mg/kg/day.

Study	Postnatal Exposure Days	Postnatal Effect Days ^1^	Critical Observed Effect	LOAEL or NOAEL (mg/kg/day) ^2^
Kern et al., 2010 [14]	1–21	33-46	Decreased learning capacity (increase use of stereotyped response strategy) ^3^	25 (LOAEL)
Kern et al., 2011 [51]	1–21	24 ^4^	Increased expression of glial acid protein in the prefrontal cortex ^5^	25 (LOAEL)
Beaudin et al., 2013 [52]	1–21	120–150 ^6^	Decreased fine sensorymotor function ^7^	25 (NOAEL)
	1–400			25 (LOAEL)

^1^ Lifestage where the observed effect has been measured. ^2^ *n* = 20 rats per dose. ^3^ This strategy consisted in finding food hidden at extremities of an 8-arms labyrinth by systematically moving from one adjacent arm to the other. ^4^ *n* = 16 to 24 rats per dose. ^5^ Results in an astrocytes activation, which constitutes a sign of neurological inflammation. ^6^ *n* = 11 or 12 rats per dose. ^7^ Consist into seasing and eating food granules during the staircase test.

**Table 3:** Comparison of drinking water guidelines proposed by various public health institutions, including the one derived by the INSPQ.

Characteristics	Public Health Institution				
	Health Canada [19]	US EPA [12]	WHO [1]	MDH [13]	INSPQ [18]
Year of publication	2016	2004	2017	2012	2017
Critical studies, (specie)	[14,51,52], (rats)	[46,47,67], (human)	[2], (human)	[14], (rats)	[14,51,52], (rats)
POD, mg/kg/day (Type)	25 (LOAEL)	0.14 (NOAEL)	0.183 (NOAEL)	25 (LOAEL)	25 (LOAEL)
Composite UF, (detailed) ^2^	1000, (10 × 10 × 1× 10 × 1)	3, (3 × 1 × 1 × 1 × 1)	3, (3 × 1 × 1 × 1 × 1)	300, (10 × 10 × 1 × 3 × 1)	450, (5 × 10 × 1 × 3 × 3)
TRV, µg/kg/day	25	47	61	83	55
BW, kg	7	70	60	-	6.7
RSC, %	50	20	20	50	20
ING, L/kgBW/day ^3^	0.107	0.029	0.033	0.29	0.182
Guideline value, mg/L	100	300	400	100 ^1^	60

Abreviations: BW, body weight; ING, daily ingestion rate; POD, point of departure; RSC, relative source contribution; TRV, toxicological reference value; UF, uncertainty factor. ^1^ DWG applied to infants less than one year old. ^2^ UF_H_ × UF_A_ × UF_SC_ × UF_L_ × UF_DB_, see text for meaning. ^3^ The body-weight adjusted ingestion rates are obtained by dividing the ingestion rate, in L/day, by the BW, in Kg, that are chosen by each institutions in the calculation of their respective drinking water guideline.

## Supplementary Materials

The following are available online at http://www.mdpi.com/1660-4601/15/6/1293/s1, Table S1. Total blood concentrations (mean, standard derivation and 95th percentiles) of Mn measured in presumably sensitive human subpopulations as reported in other studies. I. Consideration regarding the application of 3-fold UF_DB_ to account for the fact that some LOAEL values lower than the POD retained in the current assessment are available in the literature. II.Considerations with regard to the choice of the appropriate BW-adjusted drinking water ingestion rate.
