# Supplementary material for: Deriving A Drinking Water Guideline for A Non-Carcinogenic Contaminant: The Case of Manganese
Source: Int J Environ Res Public Health. 2018 Jun 20;15(6):1293. doi: 10.3390/ijerph15061293 (PMC6025359; doi:10.3390/ijerph15061293)
Supplement: Supplementary file 1 [file ijerph-15-01293-s001.pdf]

Supplemental Materials

Deriving A Drinking Water Guideline for A Non-Carcinogenic Contaminant: The Case of Manganese

Mathieu Valcke [1,2], Marie-Hélène Bourgault [1], Sami Haddad [2], Michèle Bouchard [2], Denis Gauvin [1] and Patrick Levallois [1,3,*]

[1] Direction de la Santé Environnementale et de la Toxicologie, Institut National de Santé Publique du Québec, 945 Avenue Wolfe, Québec, QC G1V 5B3, Canada; mathieu.valcke@inspq.qc.ca (M.V.); marie-helene.bourgault@inspq.qc.ca (M.-H.B.); denis.gauvin@inspq.qc.ca (D.G.)
[2] Department of Environmental and Occupational health, École de Santé Publique, C.P. 6128, Succursale Centre-Ville, Montréal, QC H3C 3J7, Canada; sami.haddad@umontreal.ca (S.H.); michele.bouchard@umontreal.ca (M.B.)
[3] Department of Social and Preventive Medicine, Faculté de Médecine, Pavillon Ferdinand-Vandry, 1050 Avenue de la Médecine, local 00241, Université Laval, Québec, QC G1V 0A6, Canada
* Correspondence: patrick.levallois@msp.ulaval.ca; Tel.: +1-418-650-5115 (ext. 5216)

Table S1. Total blood concentrations (mean, standard derivation and 95th percentiles) of Mn measured in presumably sensitive human subpopulations as reported in other studies

**Population**	**Mean (μg/L)**	**Standard deviation (μg/L)**	**95th percentile**	**Ratio 95th centile/mean**	**Reference**
Neonates	40.37	11.55	59.31 [1]	1.5	[1]
Neonates	56.4	16.47	83.4 [1]	1.5	[2]
infant (1-11 mo)	26.1	18.9	57.1 [1]	2.2	[2]
Children deficient in iron	25.5	11.27	43.98 [1]	1.7	[3]
Elderly (aged 60 – 79 years)	9.4	ND	15	1.6	[4]

[1] Estimate of the 95th percentile as per the default statistical assumption for a lognormal distribution that: 95th percentile = mean + (1.64 × standard deviation)

I. *Consideration regarding the application of 3-fold UF_{DB} to account for the fact that some LOAEL values lower than the POD retained in the current assessment are available in the literature*

LOAEL values that are lower than the POD retained here have been observed in several neurodevelopmental studies held in neonate rodents. Additionally to those cited by Health Canada [5], ATSDR also report studies exhibiting LOAEL values lower than 25 mg/kg-day [6]. These studies were reviewed here in order to bring out the rationale based on which they were excluded from Health Canada's assessment. Also, a PubMED search was performed to complement this review, using the keyword « manganese » and applying a filter in order to select only the articles published since the last version of Health Canada's Technical Document for public consultation entitled "Guidelines for Canadian Drinking Water Quality - manganese", that is January 2017. The following observations can be made on the selected articles, which are those that solely focused on manganese-exposed neonates rodents (n = 10):

1) The PubMED search allowed to retrieve only one other relevant publication, conducted by the same research group that performed the rat studies from which the POD chosen here was selected (Table 2).

Replicating the same protocol, Beaudin et al. have observed adverse attention capacity and awaken state regulation in rats exposed on PND 1-21 to 25 mg/kg/day [7] somewhat further supporting this dose as a relevant LOAEL value.

2. Among the nine other retrieved studies in which a LOAEL lower than 25 mg/kg/day is reported, 7 showed significant weaknesses, namely the use of a single exposure dose [8,9], the measure of solely biochemical parameters [10,11], the exposure doses not being reported in mg/kg/day by the authors [12,13], the lack of statistical test [14].

3) The remaining two studies from Dorman et al. [15] and Brenneman et al. [16] used similar protocols as those followed by the authors indicated in Table 2. Indeed, pup rats received manganese chloride ($MnCl_2{\bullet}4H_2O$) *per os* on PND 1-21 at exposure doses of 25 and 50 mg of $MnCl_2{\bullet}4H_2O$, which corresponds to manganese doses of respectively 11 and 22 mg/kg/day.

4) The only statistically significant effect observed by Dorman et al. (n = 20 pup rats per dose) is an increase, at exposure dose = 11 mg/kg/day, of the maximal amplitude of the jump reaction to pulsed acoustic stimulus [15].

5) Brenneman et al., for their part, observed an increase in the spontaneous motor activity at the exposure dose of 22 mg/kg/day, but no such effect at the exposure dose of 11 mg/kg/day (n = 15 female rats per dose) [16].

The study from Dorman et al. [15] therefore suggests that the lowest LOAEL associated to adverse neurological effects in neonate rats exposed to manganese could well be 11 mg /kg/day rather than the 25 mg/kg/day chose here for POD. However, no standardized protocol supports the neurophysiological tests used by Dorman et al., nor has it been replicated or published elsewhere. [15], although they are in fact mentioned by Health Canada and MDH [17–19]. From another hand, despite the fact that Dorman et al. cannot appreciate the relevancy of their studied physiological outcome (maximal amplitude of the jump reaction to pulsed acoustic stimulus), in terms of toxicological significance, they evoke that such reflex could be modulated by dopamine. Besides, other authors suggest the existence of a link between the modulation of the reaction to pulsed acoustic stimulus and emotional state as well as attention capacity [20,21], two neurological functions that have been shown to be alterated following exposure to manganese [18,22]. Overall for these reasons, it was chosen here to apply a 3-fold UF_L to the Kern et al.'s POD of 25 mg/kg/day rather than using an alternative POD of 11 mg/kg/day without such UFL, which err to the side of slightly greater safety given that 25/3 = 8.33 mg/kg/day, is lower than Dorman et al.'s LOAEL.

II. *Considerations with regard to the choice of the appropriate BW-adjusted drinking water ingestion rate*

If the POD is based on a study in which the exposure of interest occurred through lifetime, therefore during the period of infancy deemed more susceptible to exposure to environmental contaminants, it is believed that the increased exposure rate, on a body-weight basis, during infancy has been accounted for by the experimental design. Thus, it is considered that the use of the parameters corresponding to the most exposed adults, namely at the 95th percentile value, is protective enough for the entire population. This value has been estimated by the INSPQ for Quebec population [23] to 44 mL/kg BW/day, which corresponds to 3.08 L/day IR value for an adult's 70 kg BW value.

If the POD is based on a study in which the exposure of interest occurred solely during adulthood, the increased exposure rate during infancy has not been accounted for during the experimental setting. To compensate this gap, body-weight adjusted daily ingestion rate of drinking water weighted over the first 7 years of life is used. This duration is considered as the smallest chronic exposure duration as per US EPA's drinking water guidelines and health advisories [24] , and therefore appears relevant to the derivation of a

chronic exposure guideline such as the one derived here. Based on data for the Quebec children aged 0-6 months, 0.5 - < 5 years, and 5 - < 12 years old [23], the 7-years weighted mean body-weight value is 18.7 kg whereas in the case of drinking water ingestion rate, the 95th percentile value, chosen to reflect high drinking water consumers, is 1.95 L/day (or 104 mL/kg BW/day). Finally, if the POD has been determined for an exposure that occurred during a period corresponding to a specific window of susceptibility, the mean BW and 95th percentile value of ingestion rate that are being used are those that correspond to this specific time period. Since it would account for the individuals exposed during the most sensitive period of their life, it accounts de facto for the concern of protecting the most sensitive individuals, mentioned above.

References

1. Spencer, A. Whole blood manganese levels in pregnancy and the neonate. *Nutr. Burbank Los Angel. Cty. Calif* **1999**, *15*, 731–734.
2. Mizoguchi, N.; Nishimura, Y.; Ono, H.; Sakura, N. Manganese elevations in blood of children with congenital portosystemic shunts. *Eur. J. Pediatr.* **2001**, *160*, 247–250.
3. Park, S.; Sim, C.-S.; Lee, H.; Kim, Y. Blood Manganese Concentration is Elevated in Infants with Iron Deficiency. *Biol. Trace Elem. Res.* **2013**, *155*, 184–189, doi:10.1007/s12011-013-9782-9.
4. Health Canada. *Second Report on Human Biomonitoring of Environmental Chemicals in Canada* Available online: https://www.canada.ca/en/health-canada/services/environmental-workplace-health/reports-publications/environmental-contaminants/second-report-human-biomonitoring-environmental-chemicals-canada-health-canada-2013.html (accessed on Apr 19, 2018).
5. Health Canada. *Manganese in Drinking Water. Document for Public Consultation Prepared by the Federal-Provincial-Territorial Committee on Drinking Water Consultation period ends August 5, 2016.*; 2016;
6. *ATSDR Toxicological Profile: Manganese* Available online: https://www.atsdr.cdc.gov/ToxProfiles/tp.asp?id=102&tid=23 (accessed on Sep 20, 2017).
7. Beaudin, S. A.; Strupp, B. J.; Strawderman, M.; Smith, D. R. Early Postnatal Manganese Exposure Causes Lasting Impairment of Selective and Focused Attention and Arousal Regulation in Adult Rats. *Environ. Health Perspect.* **2017**, *125*, 230–237, doi:10.1289/EHP258.
8. Chandra, S. V.; Shukla, G. S.; Saxena, D. K. Manganese-induced behavioral dysfunction and its neurochemical mechanism in growing mice. *J. Neurochem.* **1979**, *33*, 1217–1221.
9. McDougall, S. A.; Reichel, C. M.; Farley, C. M.; Flesher, M. M.; Der-Ghazarian, T.; Cortez, A. M.; Wacan, J. J.; Martinez, C. E.; Varela, F. A.; Butt, A. E.; Crawford, C. A. Postnatal manganese exposure alters dopamine transporter function in adult rats: Potential impact on nonassociative and associative processes. *Neuroscience* **2008**, *154*, 848–860, doi:10.1016/j.neuroscience.2008.03.070.
10. Deskin, R.; Bursian, S. J.; Edens, F. W. Neurochemical alterations induced by manganese chloride in neonatal rats. *Neurotoxicology* **1981**, *2*, 65–73.
11. Kontur, P. J.; Fechter, L. D. Brain manganese, catecholamine turnover, and the development of startle in rats prenatally exposed to manganese. *Teratology* **1985**, *32*, 1–11, doi:10.1002/tera.1420320102.
12. Tran, T. T.; Chowanadisai, W.; Crinella, F. M.; Chicz-DeMet, A.; Lönnerdal, B. Effect of high dietary manganese intake of neonatal rats on tissue mineral accumulation, striatal dopamine levels, and neurodevelopmental status. *Neurotoxicology* **2002**, *23*, 635–643.
13. Tran, T. T.; Chowanadisai, W.; Lönnerdal, B.; Le, L.; Parker, M.; Chicz-Demet, A.; Crinella, F. M. Effects of neonatal dietary manganese exposure on brain dopamine levels and neurocognitive functions. *Neurotoxicology* **2002**, *23*, 645–651.
14. Shukakidze, A.; Lazriev, I.; Mitagvariya, N. Behavioral impairments in acute and chronic manganese poisoning in white rats. *Neurosci. Behav. Physiol.* **2003**, *33*, 263–267.
15. Dorman, D. C.; Struve, M. F.; Vitarella, D.; Byerly, F. L.; Goetz, J.; Miller, R. Neurotoxicity of manganese chloride in neonatal and adult CD rats following subchronic (21-day) high-dose oral exposure. *J. Appl. Toxicol. JAT* **2000**, *20*, 179–187.
16. Brenneman, K. A.; Cattley, R. C.; Ali, S. F.; Dorman, D. C. Manganese-induced developmental neurotoxicity in the CD rat: is oxidative damage a mechanism of action? *Neurotoxicology* **1999**, *20*, 477–487.

17. Beaudin, S. A.; Nisam, S.; Smith, D. R. Early life versus lifelong oral manganese exposure differently impairs skilled forelimb performance in adult rats. *Neurotoxicol. Teratol.* **2013**, *38*, 36–45, doi:10.1016/j.ntt.2013.04.004.
18. Kern, C. H.; Smith, D. R. Preweaning Mn exposure leads to prolonged astrocyte activation and lasting effects on the dopaminergic system in adult male rats. *Synap. N. Y. N* **2011**, *65*, 532–544, doi:10.1002/syn.20873.
19. Kern, C. H.; Stanwood, G. D.; Smith, D. R. Preweaning manganese exposure causes hyperactivity, disinhibition, and spatial learning and memory deficits associated with altered dopamine receptor and transporter levels. *Synap. N. Y. N* **2010**, *64*, 363–378, doi:10.1002/syn.20736.
20. Le Duc, J. *Modulation du réflexe acoustique de sursaut et de l'inhibition par le prépulse : Une comparaison entre les jeunes adultes et les âgés*, Université de Montréal, Département de Sciences biomédicales Faculté de Médecine: Montréal, QC, Canada 2013.
21. Pålsson, E.; Söderlund, G.; Klamer, D.; Bergquist, F. Noise benefit in prepulse inhibition of the acoustic startle reflex. *Psychopharmacology (Berl.)* **2011**, *214*, 675–685, doi:10.1007/s00213-010-2074-6.
22. Bauer, J. A.; Claus Henn, B.; Austin, C.; Zoni, S.; Fedrighi, C.; Cagna, G.; Placidi, D.; White, R. F.; Yang, Q.; Coull, B. A.; Smith, D.; Lucchini, R. G.; Wright, R. O.; Arora, M. Manganese in teeth and neurobehavior: Sex-specific windows of susceptibility. *Environ. Int.* **2017**, *108*, 299–308, doi:10.1016/j.envint.2017.08.013.
23. INSPQ *Lignes directrices pour la réalisation des évaluations du risque toxicologique d'origine environnementale au Québec. Institut national de santé publique du Québec.*; Institut national de santé publique du Québec: Québec, QC, Canada 2012.
24. U.S. EPA *2012 Edition of the Drinking Water Standards and Health Advisories*; U.S. Environmental Protection Agency: Washington, D.C., USA, 2012.
